# CSM‐peptides: A computational approach to rapid identification of therapeutic peptides

**DOI:** 10.1002/pro.4442

**Published:** 2022-09-28

**Authors:** Carlos H. M. Rodrigues, Anjali Garg, David Keizer, Douglas E. V. Pires, David B. Ascher

**Affiliations:** ^1^ Structural Biology and Bioinformatics, Department of Biochemistry University of Melbourne Melbourne Victoria Australia; ^2^ Systems and Computational Biology, Bio21 Institute, University of Melbourne Melbourne Victoria Australia; ^3^ Computational Biology and Clinical Informatics Baker Heart and Diabetes Institute Melbourne Victoria Australia; ^4^ School of Chemistry and Molecular Biosciences University of Queensland St Lucia Queensland Australia; ^5^ School of Computing and Information Systems University of Melbourne Melbourne Victoria Australia

**Keywords:** machine learning, peptide screening, therapeutic peptides, web server

## Abstract

Peptides are attractive alternatives for the development of new therapeutic strategies due to their versatility and low complexity of synthesis. Increasing interest in these molecules has led to the creation of large collections of experimentally characterized therapeutic peptides, which greatly contributes to development of data‐driven computational approaches. Here we propose CSM‐peptides, a novel machine learning method for rapid identification of eight different types of therapeutic peptides: anti‐angiogenic, anti‐bacterial, anti‐cancer, anti‐inflammatory, anti‐viral, cell‐penetrating, quorum sensing, and surface binding. Our method has shown to outperform existing approaches, achieving an AUC of up to 0.92 on independent blind tests, and consistent performance on cross‐validation. We anticipate CSM‐peptides to be of great value in helping screening large libraries to identify novel peptides with therapeutic potential and have made it freely available as a user‐friendly web server and Application Programming Interface at https://biosig.lab.uq.edu.au/csm_peptides.

## 1 INTRODUCTION

Peptides are versatile molecules that play essential roles in signaling processes, such as growth factors, neurotransmitters, and anti‐infectives. Given their lower complexity of synthesis and production costs compared to traditional protein‐based drugs, peptides are attractive candidates for developing new therapeutics and diagnostics.^1^ An increasing number of peptides have been identified with a wide variety of therapeutic applications, including treatments for cancer,^2^ inflammatory diseases^3^ and as drug delivery mechanisms.^4^ Despite these efforts, experimental screening of novel peptides remains a time consuming and expensive endeavor.

Several computational methods have been proposed to help identify and characterize the functional mechanisms of peptides more efficiently^4^, ^5^, ^6^, ^7^, ^8^, ^9^, ^10^; however, despite these relevant efforts, available approaches present variable performance and lack of easy‐to‐use interfaces, limiting their use to those with specialist knowledge in addition to not providing mechanisms to facilitate integration within bioinformatics pipelines.

**FIGURE 1:**
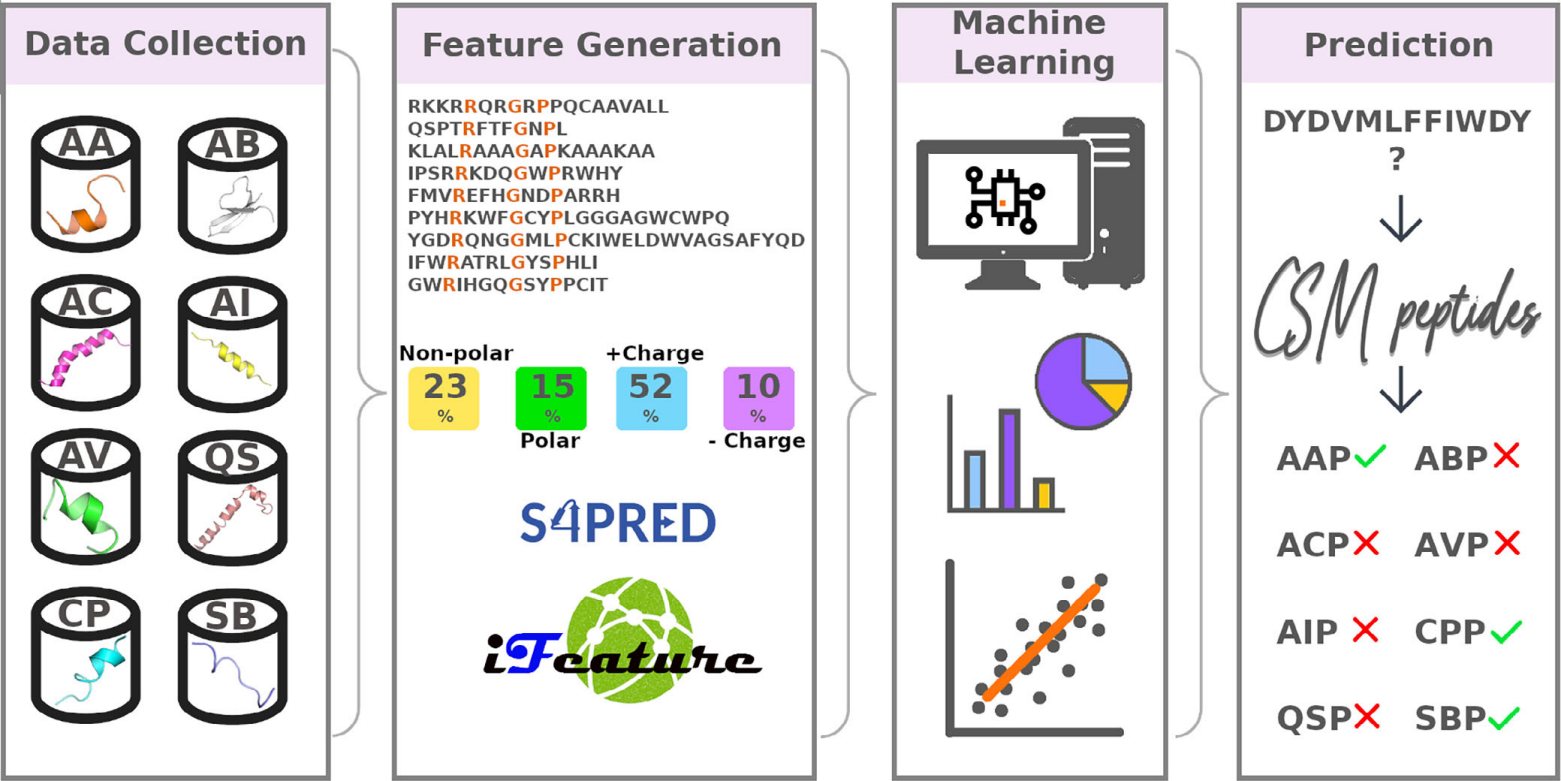
Methodology workflow for CSM‐peptides. The development of CSM‐peptides can be divided into four main steps: (1) data are collected from the literature for eight different classes of therapeutic peptides; (2) features are calculated, including evolutionary scores from substitution tables, physicochemical and indexes calculated based on each peptide sequence and predicted proportion of secondary structure; (3) feature selection and model training for each peptide class separately; (4) best performing models are deployed into a webserver and API publicly available

Here we expand our cutoff scanning matrix (CSM) platform by proposing CSM‐peptides, a novel suite of machine learning (ML) approaches to identify therapeutic peptides for eight different classes: anti‐angiogenic (AAP), anti‐bacterial (ABP), anti‐cancer (ACP), anti‐inflammatory (AIP), anti‐viral (AVP), cell‐penetrating (CPP), quorum sensing (QSP), and surface binding (SBP). Our method explores physicochemical properties and incorporates predictions of secondary structure and disorder regions of peptide sequences (Figure 1), which we show achieves equivalent or better performance than alternative approaches. CSM‐peptides is a user‐friendly web resource that can be easily incorporated into analytical pipelines via an Application Programming Interface (API) at https://biosig.lab.uq.edu.au/csm_peptides.

## 2 RESULTS AND DISCUSSION

### 2.1 Common properties of active peptides

Overall peptide length varied from 5 to 97 amino acids long, with peptides from classes ACP and ABP showing the highest average values among all other classes (22 and 30 amino acids long, respectively). QSP peptides were shown to be the shortest with average length of 11 amino acids long. This could be related to their role in rapid signaling response in the cell, leading to the synthesis of less complex signal molecules. In addition, at a neutral pH, net charge for ACP, ABP, and CPP presented the highest values ranging from 3.6 to 5.1, while QSP and SBP showed values closer to 0. General physicochemical properties for all peptide classes are summarized in Table S1 in the Supplementary Material.

In terms of amino acid composition, for all peptide classes, on average non‐polar and positively charged amino acids were enriched, including Glycine (G), Lysine (K), Arginine (R), and Leucine (L), as opposed to negatively charged amino acids, such as D and E, which showed low proportions across the eight different classes of peptides investigated in this study. The latter has been shown to be an expected characteristic of these molecules since negatively charged amino acids may interfere during the course of interaction with an also negatively charged cell membrane.^6^ A comprehensive summary of the proportion of the 20 standard amino acid residues for each peptide class is displayed in Figure S1 and stratification by residue type (polar, non‐polar, aromatic, and charged) is available in Table S2 in Supplementary Materials.

### 2.2 Predicting therapeutic peptides

After performing our greedy stepwise approach to feature selection for binary classifiers for each peptide class separately, the number of selected features per model varied from 21 (AIP) to 93 (ABP). We observed, however, that features representing physicochemical properties were consistent across all peptide classes, most notably those representing distributions of amino acid attributes (e.g., hydrophobicity, charge, polarity, and solvent exposure) were used by all models. In addition, BLOSUM indices were selected by almost all predictive models. Interestingly, features accounting for secondary structure type and disorder regions were only selected together for the ABP class, and separately for models built for the AVP and CPP classes. Feature importances for each peptide class are summarized in Tables S3–S10. Overall, the importance of features was evenly spread for predictors of all classes, except AIP and QSP, in which the percentage of charged residues, calculated via amino acid composition‐transition‐distribution (CTD)^11^ using iFeature, accounted for nearly 25% importance. Amino acid CTD features have been previously shown to be an important variable for predicting CPP,^12^ and in this study, this property was present for nearly all other binary classifiers with a more moderate contribution to the final predictive models, including CPP.

Performance on training for all eight predictive models, using their respective final set of selected features, was assessed under 10‐fold CV and results have been summarized in Table S11 in the Supplementary Materials. Overall, performances in terms of AUC were robust with values ranging from 0.83 for AIP to 0.99 for ABP. A closer inspection of true positive rate (TPR; sensitivity) and true negative rate (TNR; specificity) indicate that predictive models for classes AAP and SBP showed the highest discrepancy between the two metrics, while the rest remained consistent in their ability to correctly identify peptides of these classes from others. We believe the lower agreement between TPR and TNR on training for the AAP and SBP sets (206 and 160, respectively) to be related to the low number of entries available for these two classes, limiting the outcomes of the machine learning algorithms evaluated. Surprisingly, the AIP class, which has the largest number of entries for training, showed similar performance on training from AAP and SBP, suggesting that these molecules have a more complex mechanism of action and would benefit from alternative methods for encoding protein sequence information.

### 2.3 Comparison with alternative methods

**FIGURE 2:**
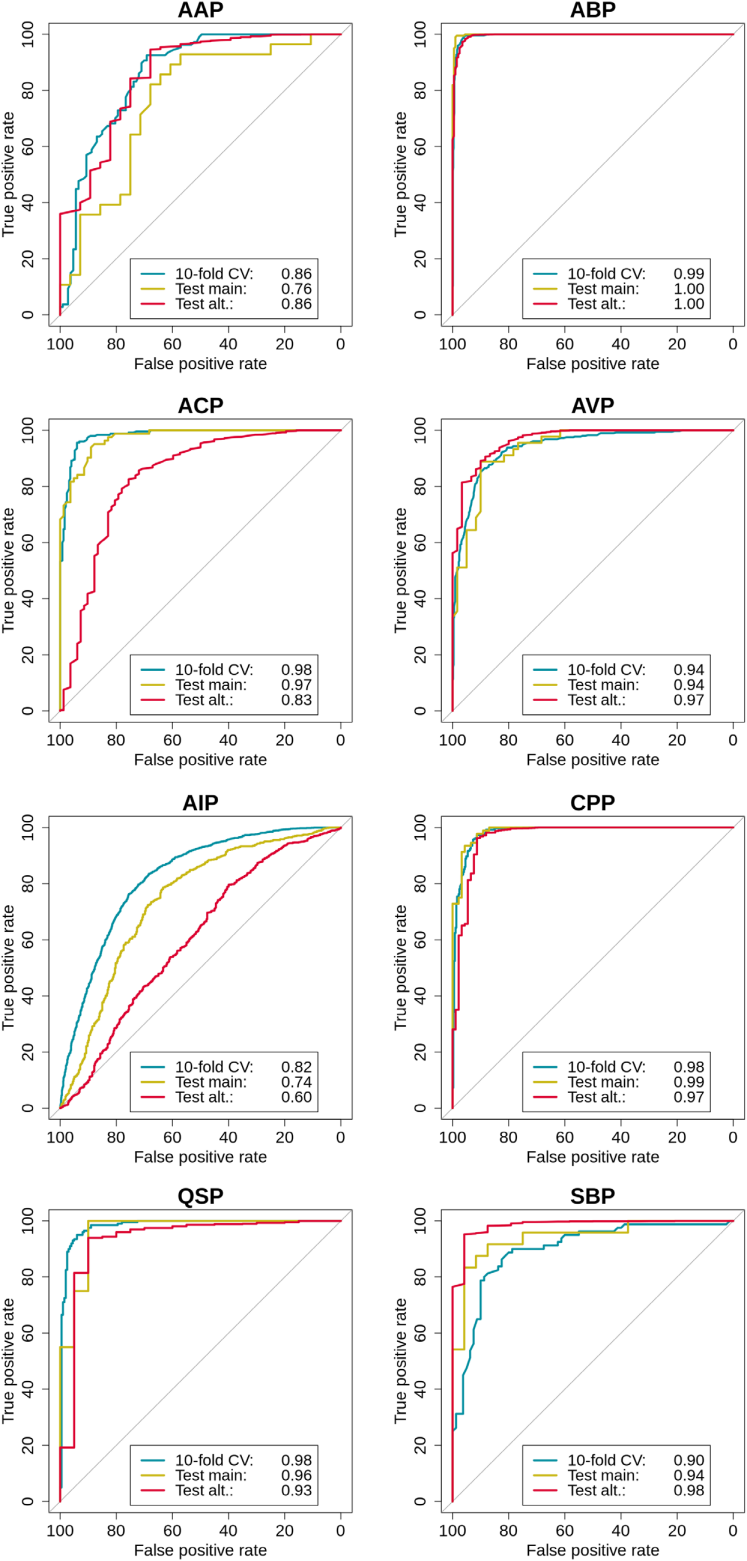
Performance of CSM‐peptides on 10‐fold CV and two independent blind‐tests for predictive models for eight classes of therapeutic peptides. Results are shown as ROC curves where green lines represent results on 10‐fold CV, yellow and red describe results of assessing the performance on main and alternative test sets, respectively. AAP, anti‐angiogenic; ABP, anti‐bacterial; ACP, anti‐cancer; AIP, anti‐inflammatory; AVP, anti‐viral; CPP, cell‐penetrating; QSP, quorum sensing; SBP, surface binding

Our peptide specific predictive models were further assessed over two independent datasets and outcomes compared with those reported on the PEPred study^9^ and based on the results output from the PPTPP tool.^10^ CSM‐peptides outperformed both methods on the SBP class, achieving an AUC of 0.94 on the main test set and 0.98 on the alternative set (Figure 2). For CPP and ACP classes, our method outperformed both PPTPP and PEPpred on the alternative test set and achieved similar performance on the main test sets. Interestingly, all methods had a drop in performance for the AIP class corroborating our previous assumption that, despite using distinct modeling techniques to encode and select features, solely representing physicochemical properties are insufficient for machine learning approaches which may underfit, possibly due to a more complex mechanism of action for this class of peptides,^13^ also corroborated by the low number of features selected. In addition, we have compared the performance of CSM‐peptides for classes ACP and AVP with AI4ACP^14^ and FIRM‐AVP,^15^ respectively. In both cases, the class specific predictors from our approach showed superior performance when compared with the alternative methods for the two blind tests used in this work. Interestingly, performance on the alternative test set showed a considerable drop in terms of Matthews correlation coefficient (MCC) and TNR, indicating that more negative entries are being misclassified. These results may be explained by the quality and amount of experimental data available for most peptide classes, such as AAP, QSP, and SBP with a total of 214, 400, and 160 entries used for training, respectively. We hypothesize this limitation to be the main cause for preventing most algorithms to explore the search space properly for the majority of classes, and consequently limiting their ability to correctly distinguish between positive and negative samples. This trend is more evidently observed in such a diversified set of negative samples as available in the alternative test set.

Performances on both independent test sets are summarized in Table 1 for all methods. PEPred results are reported in terms of AUC as this is the only metric reported in the study and at the time of writing this manuscript the server is down and neither the source code for local installation is available for installation. Furthermore, given the limitation in terms of length of peptides which could be analyzed by AI4ACP (≤50 amino acid long), the results for this method are reported after removing such entries from main and alternative test sets.

**TABLE 1:** Performance comparison of CSM‐peptides with other methods on two independent test sets for predictive models of each therapeutic peptide class

Class	Method	Main test set				Alternative test set			
		AUC	TPR	TNR	MCC	AUC	TPR	TNR	MCC
AAP	CSM‐peptides	0.76	0.57	0.92	0.53	0.86	0.67	0.86	0.18
	PPTPP	0.77	0.71	0.78	0.50	0.75	0.71	0.70	0.10
	PEPred	0.80	–	–	–	0.77	–	–	–
ABP	CSM‐peptides	1.00	0.98	0.99	0.97	1.00	0.96	0.98	0.90
	PPTPP	0.98	0.92	0.96	0.89	0.96	1.00	0.93	0.61
	PEPred	0.97	–	–	–	0.96	–	–	–
ACP	CSM‐peptides	0.97	0.90	0.90	0.80	0.83	0.87	0.50	0.15
	PPTPP	0.87	0.80	0.81	0.62	0.71	0.80	0.38	0.07
	PEPred	0.94	–	–	–	0.63	–	–	–
	AI4ACP	0.49	0.88	0.1	−0.02	0.5	0.15	0.85	0.00
AIP	CSM‐peptides	0.71	0.43	0.93	0.43	0.54	0.67	0.35	0.02
	PPTPP	0.70	1.00	0.04	0.15	0.39	0.08	0.83	−0.08
	PEPred	0.75	–	–	–	0.63	–	–	–
AVP	CSM‐peptides	0.94	0.90	0.86	0.76	0.97	0.96	0.74	0.27
	PPTPP	0.96	0.91	0.77	0.70	0.90	0.91	0.47	0.13
	PEPred	0.94	–	–	–	0.95	–	–	–
	FIRM‐AVP	0.67	0.90	0.44	0.20	0.51	0.30	0.72	0.01
CPP	CSM‐peptides	0.99	0.90	0.96	0.87	0.97	0.85	0.98	0.78
	PPTPP	0.96	0.86	0.88	0.75	0.85	0.86	0.55	0.17
	PEPred	0.95	–	–	–	0.87	–	–	–
QSP	CSM‐peptides	0.98	0.90	0.95	0.85	0.94	0.95	0.87	0.24
	PPTPP	0.94	0.80	1.00	0.81	0.85	0.75	0.77	0.122
	PEPred	0.96	–	–	–	0.89	–	–	–
SBP	CSM‐peptides	0.94	0.83	0.91	0.75	0.98	0.87	0.97	0.51
	PPTPP	0.77	0.75	0.66	0.41	0.84	0.66	0.87	0.17
	PEPred	0.67	–	–	–	0.79	–	–	–

*Note*: Results are shown in terms of area under the ROC curve (AUC), sensitivity (TPR), specificity (TNR) and Matthew's correlation coeff (MCC). Cells filled with a dash (−) indicate cases where results were not available or could not be generated.

Abbreviations: AAP, anti‐angiogenic; ABP, anti‐bacterial; ACP, anti‐cancer; AIP, anti‐inflammatory; AVP, anti‐viral; CPP, cell‐penetrating; QSP, quorum sensing; and SBP, surface binding.

Given the high performance observed for most peptide classes when predicting the test sets, we evaluated the level of contamination between training and test sets for each peptide class using three different cutoffs of similarity (Table S12). Here we used the SequenceMatcher module, available in the *difflib* Python package, similarly to what has been described in a previous study for clustering peptide sequences.^16^ Except for the AVP and QSP classes, all entries in the test set are non‐redundant to the train set when using a cutoff of 75% similarity for all peptide classes. Using a threshold of 85% similarity, the AVP class was the only class with a small number of peptides, which differ only by one amino acid from a single entry in the training set. After removing this entry from the training set and re‐training the predictive model for this class no significant drop in performance was observed.

Finally, additional non‐redundant peptides were retrieved from DRAMP^17^ for the ABP, ACP, and AVP classes, comprising 3,019, 176, and 132 peptides respectively. CSM‐peptides achieved accuracies ranging from 61% on ACP class to 83% on ABP (Table S13).

### 2.4 Web server

Users can query the website using a single peptide sequence or providing a list of sequences in FASTA format for batch processing (Figure S2A) via the upload option. Examples and format descriptions are available both on the submission page and the help page via the top navigation menu. If an email is provided, the user will be notified of the results when they finish processing.

The output page presents the results as a downloadable table (Figure S2B), where each row summarizes the output for all eight binary classifiers for each peptide class (AAP, ABP, ACP, AIP, AVP, CPP, QSP, and SBP) for a particular entry. A probability score is shown upon hovering the mouse cursor over the predicted label for a given class. Here we used the default cut‐off of >0.5 to define the final predicted label as positive. In addition, a “Detail” button is available for each entry to assist users when comparing general physicochemical properties of a given input peptide with the overall class distribution. A detailed description with examples on how to run predictions is available in the help page, and additional documentation for querying the web server using the API is available at https://biosig.lab.uq.edu.au/csm_peptides/docs.

## 3 CONCLUSION

Here we presented CSM‐peptides, a web platform for characterizing peptide sequences for eight different classes of therapeutic peptides. Our approach integrates a diverse range of physicochemical properties and sequence‐based properties tailored in individual predictive models for each peptide class via supervised learning. Overall, CSM‐peptides shows equivalent or superior performance over most recent approaches on the same blind test, and robust accuracies on an independent set of peptides for classes ABP, ACP, and AVP. More in‐depth research into classes of therapeutic peptides with a more complex mode of action, such as AIP, is still needed, as well as the quality and availability of experimentally determined positive and negative peptides for the development of more generalizable methods. Furthermore, alternative deep learning and natural language processing (NLP) methods may represent an attractive venue for encoding peptide sequences.

We anticipate CSM‐peptides to be of great value to the scientific community for the study of therapeutic peptides and for a more rapid and effective screening and characterization of novel peptide sequences. Our method includes an API to assist more experienced users when integrating our predictions into their research analysis pipelines, and it is also freely available as a user‐friendly and easy‐to‐use server at https://biosig.lab.uq.edu.au/csm_peptides.

## 4 MATERIALS AND METHODS

### 4.1 Datasets

Experimentally characterized peptides with activity for eight different classes (AAP, ABP, ACP, AIP, AVP, CPP, QSP, and SBP) were collected from previous studies.^4^, ^5^, ^6^, ^7^, ^8^, ^18^, ^19^, ^20^ Negative samples comprised entries without experimental evidence for a respective class. Data were divided into training and main test sets following an 80/20 split with a balanced proportion between positive/negative entries, except for the AIP and AVP classes where there was an imbalance toward negative entries. In addition, an alternative non‐redundant test set was used to further validate the models, using the same set of positive entries. Given the lack of negative samples for each peptide class available in the literature, we generated 2,000 negative entries for each class of peptides using two approaches that have been broadly implemented on for sequence‐based predictors of peptide activity^21^, ^22^, ^23^, ^24^, ^25^: (1) randomly shuffling sequences from the positive class, which is based on the hypothesis that the possibility of generating an active peptide from a random sequence is very low^26^; and (2) random peptides with no activity for any of the eight classes were extracted from Swiss‐Prot.^27^ Proportions of positive and negative samples for each peptide class on training and independent test sets are summarized in Table S14.

### 4.2 Feature generation and machine learning

For each peptide, scores from amino acid substitution matrices were extracted from the AAINDEX database^28^ and additional properties calculated using iFeature^29^ and Peptides package,^30^ including amino acid composition, interaction potential scores (summarized in Table S15), and BLOSUM indices derived from physicochemical properties that have been subjected to a VARIMAX analysis and an alignment matrix of the 20 standard AAs using the BLOSUM62 matrix.^31^ As the AAINDEX properties are dependent on amino acid sequence length, here we calculated average and variance values for each of the 531 scores available for each peptide sequence. The proportion of secondary structures (helices, sheets, and loops) were generated using S4PRED,^32^ a novel deep semi‐supervised learning framework for predicting secondary structure components from protein sequences. Finally, in order to incorporate a broader range of features to be explored by the machine learning algorithms, we also included calculations for the proportion of intrinsically disordered regions using IUPred2A.^33^

Prior to training the predictive models, and to counterbalance the large number of features generated via AAINDEX (1,174) and iFeature (1,593), we first removed low discriminative features (properties with mostly identical values for all entries, e.g., all zeros) by applying the *VarianceThreshold* filter, available on the Scikit‐Learn library,^34^ to select only features with a variance >0.1. Feature selection was then carried out using a greedy stepwise approach^35^, ^36^ independently for each ML algorithm, where for each feature, the performance on 10‐fold cross‐validation (CV) is evaluated against the target value, using Matthews Correlation Coefficient (MCC). The best performing feature is then selected and fixed in a group of selected features. The process is repeated for each of the remaining features in combination with the previously fixed one in order to find the best pair. The procedure continues until all features are selected. The best performing subset of features are then used for training the final predictive models.

Predictive models were built for three different algorithms (ExtraTrees, GradientBoosting, and XGBoost) and the final models were selected based on performance on 10‐fold CV after feature selection. Feature importance was assessed based on importance scores measured as the total reduction of the criterion brought by the feature, namely Gini importance, which is commonly used for tree‐based algorithms to assist with model interpretability. Performance of predictive models was assessed based on a variety of metrics, including F1‐score, MCC, area under the receiving operator curve (AUC), sensitivity (TPR), and specificity (TNR). Final models were also evaluated against two non‐redundant test sets.

### 4.3 Web server

CSM‐peptides is implemented as a freely available user‐friendly web server. The server front‐end is developed using the Materialize framework version 1.0.0, while the back‐end is built with Flask (version 1.0.2), a framework for web applications built on top of the Python programming language. The web server is hosted on a Linux Server running Apache2.

## AUTHOR CONTRIBUTIONS

**Carlos Rodrigues:** Data curation (equal); formal analysis (lead); investigation (equal); methodology (lead); software (lead); validation (lead); writing – original draft (lead). **Anjali Garg:** Data curation (equal); investigation (supporting); methodology (supporting). **David Keizer:** Formal analysis (supporting); validation (supporting).

## CONFLICT OF INTEREST

The authors declare no conflicts of interest.

## Supporting information


**Figure S1**. Distribution of the proportion of amino acid content across the eight classes of therapeutic peptides. *X*‐axis shows the 20 standard amino acids and the *y*‐axis represents the average proportion in which a given amino acid appears for peptides of a particular class.
**Figure S2**. CSM‐peptides web interface. (A) On the submission page, users can submit a single protein sequence or upload a list of peptide sequences as a FASTA formatted file. (B) Results are presented as a downloadable table where predictions are shown for each peptide class.
**Table S1**. Distribution of the general physicochemical properties across the eight different classes of therapeutic peptides.
**Table S2**. Distribution of amino acid types across the eight different classes of therapeutic peptides.
**Table S3**. Feature importance for the predictive model of anti‐angiogenic peptides.
**Table S4**. Feature importance for the predictive model of anti‐bacterial peptides.
**Table S5**. Feature importance for the predictive model of anti‐cancer peptides.
**Table S6**. Feature importance for the predictive model of anti‐viral peptides.
**Table S7**. Feature importance for the predictive model of anti‐inflammatory peptides.
**Table S8**. Feature importance for the predictive model of cell‐penetrating peptides.
**Table S9**. Feature importance for the predictive model of quorum sensing peptides.
**Table S10**. Feature importance for the predictive model of surface binding peptides.
**Table S11**. Performance on 10‐fold cross validation for training predictive models for eight different therapeutic peptide classes.
**Table S12**. Proportion of identical/similar peptides in training and test sets for all peptide classes. Similarity is measured using the SequenceMatcher module, available in the *difflib* Python package, under different cutoffs of similarity.
**Table S13**. Performance of predictive models for ABP, ACP, and AVP classes on blind‐test sets of peptides retrieved from DRAMP database.
**Table S14**. Distribution of training and test sets for the eight classes of peptides in the dataset. Data are divided into a training set, used to build the predictive models, and two non‐redundant test sets.
**Table S15**. Classes of features calculated for each peptide sequence in the dataset.

## Data Availability

CSM‐peptides predictive models are freely available either as a user‐friendly web interface or as an API for programmatic access at https://biosig.lab.uq.edu.au/csm_peptides. Neither login nor license is required. All data sets used to build and evaluate the predictive models for all peptide classes discussed in this work are available for download as comma‐separated files (CSV) at https://biosig.lab.uq.edu.au/csm_peptides/data.
