# Supplementary material for: CSM‐peptides: A computational approach to rapid identification of therapeutic peptides
Source: Protein Sci. 2022 Sep 28;31(10):e4442. doi: 10.1002/pro.4442 (PMC9518225; doi:10.1002/pro.4442)
Supplement: Supplementary file 1 — Figure S1. Distribution of the proportion of amino acid content across the eight classes of therapeutic peptides. X‐axis shows the 20 standard amino acids and the y‐axis represents the average proportion in which a given amino acid appears for peptides of a particular class. Figure S2. CSM‐peptides web interface. (A) On the submission page, users can submit a single protein sequence or upload a list of peptide sequences as a FASTA formatted file. (B) Results are presented as a downloadable table where predictions are shown for each peptide class. Table S1. Distribution of the general physicochemical properties across the eight different classes of therapeutic peptides. Table S2. Distribution of amino acid types across the eight different classes of therapeutic peptides. Table S3. Feature importance for the predictive model of anti‐angiogenic peptides. Table S4. Feature importance for the predictive model of anti‐bacterial peptides. Table S5. Feature importance for the predictive model of anti‐cancer peptides. Table S6. Feature importance for the predictive model of anti‐viral peptides. Table S7. Feature importance for the predictive model of anti‐inflammatory peptides. Table S8. Feature importance for the predictive model of cell‐penetrating peptides. Table S9. Feature importance for the predictive model of quorum sensing peptides. Table S10. Feature importance for the predictive model of surface binding peptides. Table S11. Performance on 10‐fold cross validation for training predictive models for eight different therapeutic peptide classes. Table S12. Proportion of identical/similar peptides in training and test sets for all peptide classes. Similarity is measured using the SequenceMatcher module, available in the difflib Python package, under different cutoffs of similarity. Table S13. Performance of predictive models for ABP, ACP, and AVP classes on blind‐test sets of peptides retrieved from DRAMP database. Table S14. Distribution of training and test sets for t [file PRO-31-e4442-s001.docx]

**SUPPLEMENTARY MATERIAL**

**CSM-peptides: a computational approach to rapid identification
 of therapeutic peptides**

Carlos H. M. Rodrigues ^1,2,3,5^, Anjali Garg ^1,2^, David Keizer ^1,2^,
Douglas E. V. Pires ^2,3,4*^, David B. Ascher ^1,2,3,5*^

^1^ Structural Biology and Bioinformatics, Department of Biochemistry, University of Melbourne, Melbourne, Victoria, Australia

^2^ Systems and Computational Biology, Bio21 Institute, University of Melbourne, Melbourne, Victoria, Australia

^3^ Computational Biology and Clinical Informatics, Baker Heart and Diabetes Institute, Melbourne, Victoria, Australia

^4^ School of Computing and Information Systems, University of Melbourne, Melbourne, Victoria, Australia

^5^ School of Chemistry and Molecular Biosciences, University of Queensland, Brisbane, Queensland, Australia

*To whom correspondence should be addressed. D.B.A. Tel: +61 90354794 and Email: [d.ascher@uq.edu.au](mailto:d.ascher@uq.edu.au); D.E.V.P. Tel: +61 3 83448185 and Email: [douglas.pires@unimelb.edu.au](mailto:douglas.pires@unimelb.edu.au).

# FIGURES


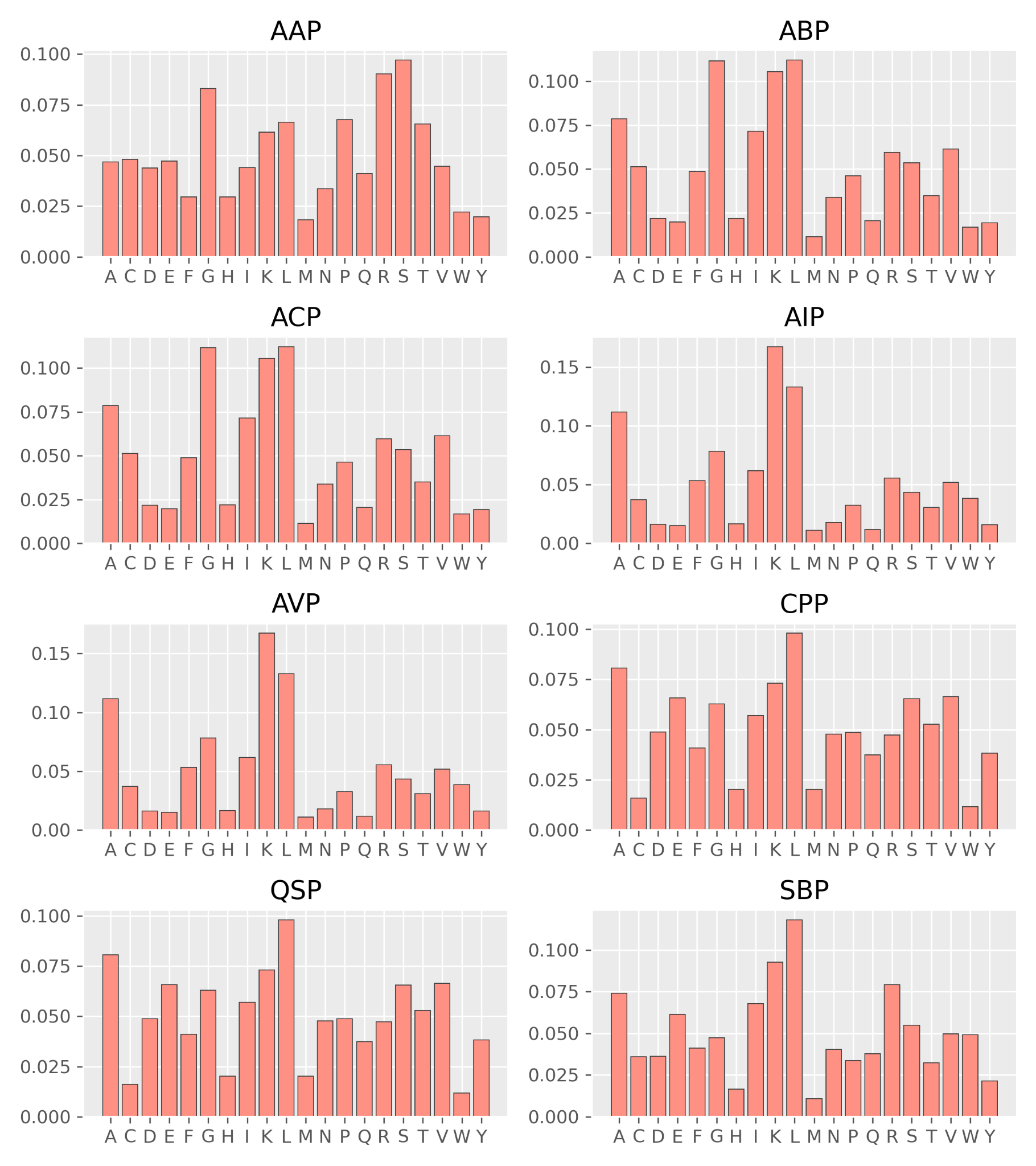


**Figure S1** - Distribution of the proportion of amino acid content across the eight classes of therapeutic peptides. X-axis shows the 20 standard amino acids and the y-axis represents the average proportion in which a given amino acid appears for peptides of a particular class.

**
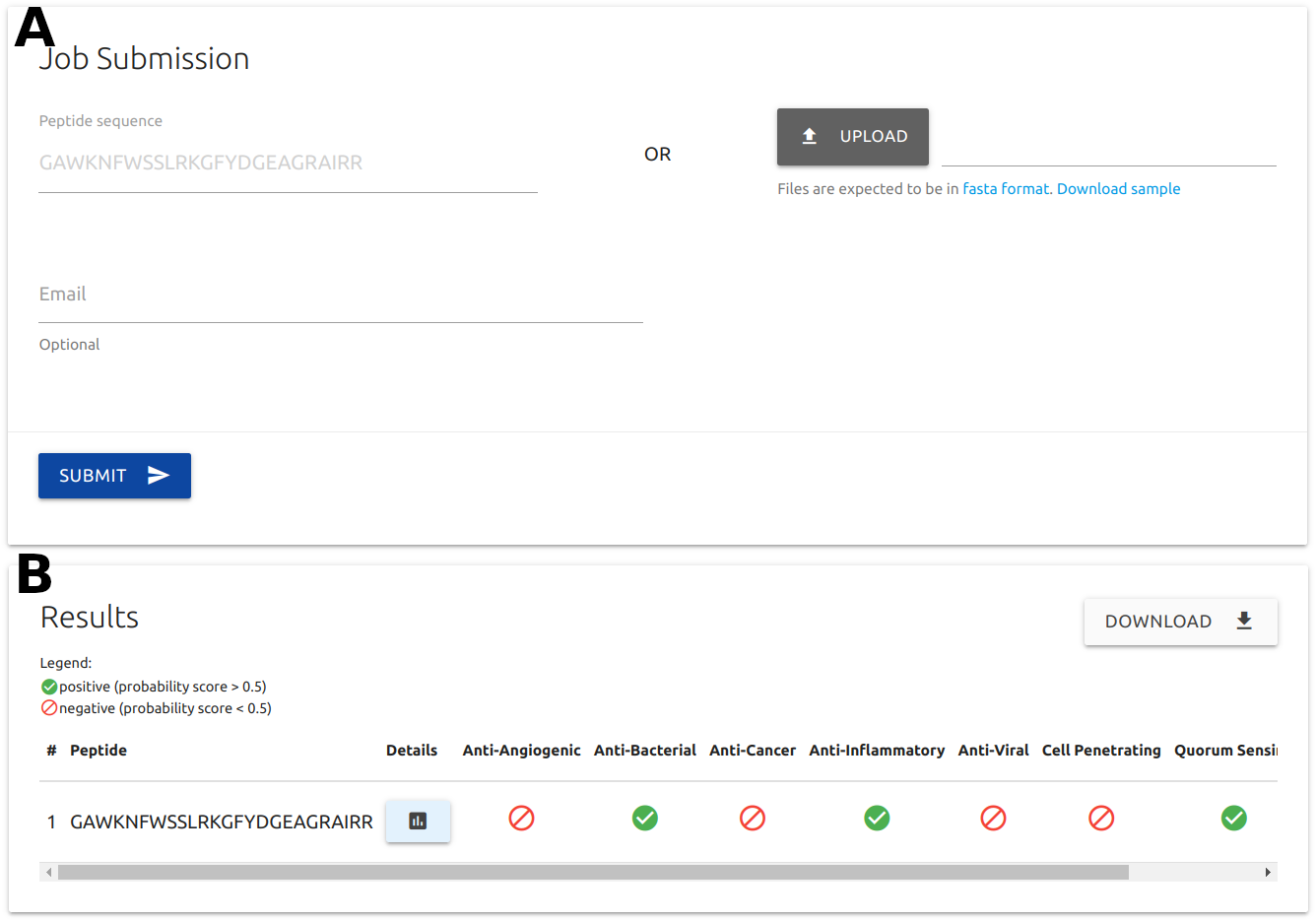
**

**Figure S2** - CSM-peptides web interface. A) On the submission page, users can submit a single protein sequence or upload a list of peptide sequences as a fasta formatted file. B) Results are presented as a downloadable table where predictions are shown for each peptide class.

#

# TABLES

**Table S1** - Distribution of the general physicochemical properties across the eight different classes of therapeutic peptides.

|  |  | **Molecular weight** | | | | **Length** | | | | **Net charge** | | | | **Hydrophobicity** | | | |
| --- | --- | --- | --- | --- | --- | --- | --- | --- | --- | --- | --- | --- | --- | --- | --- | --- | --- |
| **Class** | **count** | **avg** | **std** | **min** | **max** | **avg** | **std** | **min** | **max** | **avg** | **std** | **min** | **max** | **avg** | **std** | **min** | **max** |
| **AAP** | 107 | 2284.08 | 1021.54 | 1113.27 | 7712.88 | 20.32 | 9.24 | 11.00 | 67.00 | 1.21 | 3.07 | -7.00 | 11.99 | -0.67 | 0.87 | -2.93 | 1.75 |
| **ABP** | 800 | 3266.49 | 1522.07 | 708.55 | 11193.80 | 29.91 | 14.01 | 6.00 | 94.00 | 3.59 | 3.10 | -6.00 | 30.00 | 0.12 | 0.87 | -3.50 | 2.35 |
| **ACP** | 250 | 2451.14 | 1234.67 | 976.96 | 10587.77 | 21.93 | 11.58 | 11.00 | 97.00 | 3.79 | 3.07 | -3.91 | 12.00 | 0.10 | 0.78 | -2.25 | 1.81 |
| **AIP** | 1258 | 1917.45 | 353.87 | 1088.33 | 3012.38 | 16.97 | 2.90 | 11.00 | 25.00 | 0.11 | 2.12 | -7.99 | 12.00 | -0.27 | 0.84 | -2.73 | 3.15 |
| **AVP** | 544 | 2858.28 | 1208.25 | 711.74 | 11725.93 | 24.55 | 10.71 | 6.00 | 107.0 | 0.90 | 3.82 | -7.89 | 13.99 | -0.27 | 0.95 | -3.56 | 2.54 |
| **CPP** | 370 | 2404.10 | 980.34 | 1083.34 | 7662.82 | 20.22 | 8.40 | 10.00 | 61.00 | 5.10 | 4.54 | -6.99 | 24.00 | -0.96 | 1.17 | -4.00 | 1.65 |
| **QSP** | 200 | 1301.31 | 727.30 | 495.59 | 3403.86 | 11.26 | 6.44 | 5.00 | 30.00 | 0.69 | 1.66 | -2.00 | 7.00 | 0.10 | 1.22 | -3.14 | 3.19 |
| **SBP** | 80 | 1768.94 | 1056.19 | 678.71 | 4574.01 | 14.93 | 9.15 | 7.00 | 38.00 | 0.05 | 1.67 | -5.81 | 5.00 | -0.26 | 0.74 | -1.83 | 2.35 |

**Table S2** - Distribution of amino acid types across the eight different classes of therapeutic peptides.

|  |  | **Polar** | | | | **Non-polar** | | | | **Aromatic** | | | | **Charged** | | | |
| --- | --- | --- | --- | --- | --- | --- | --- | --- | --- | --- | --- | --- | --- | --- | --- | --- | --- |
| **class** | **count** | **avg** | **std** | **min** | **max** | **avg** | **std** | **min** | **max** | **avg** | **std** | **min** | **max** | **avg** | **std** | **min** | **max** |
| **AAP** | 107 | 10.24 | 4.75 | 3.00 | 31.00 | 10.07 | 5.68 | 2.00 | 39.00 | 2.00 | 2.04 | 0.00 | 12.00 | 5.48 | 3.60 | 0.00 | 18.00 |
| **ABP** | 800 | 11.44 | 6.74 | 0.00 | 45.00 | 18.47 | 8.72 | 0.00 | 68.00 | 3.08 | 2.41 | 0.00 | 15.00 | 6.83 | 4.22 | 0.00 | 30.00 |
| **ACP** | 250 | 8.45 | 5.88 | 1.00 | 45.00 | 13.48 | 6.60 | 4.00 | 55.00 | 2.43 | 1.69 | 0.00 | 10.00 | 5.70 | 3.48 | 0.00 | 25.00 |
| **AIP** | 1258 | 7.83 | 2.81 | 0.00 | 20.00 | 9.14 | 2.64 | 2.00 | 19.00 | 1.87 | 1.46 | 0.00 | 8.00 | 4.34 | 2.25 | 0.00 | 15.00 |
| **AVP** | 544 | 11.63 | 7.26 | 0.00 | 46.00 | 12.91 | 5.61 | 1.00 | 61.00 | 2.65 | 1.88 | 0.00 | 9.00 | 6.92 | 4.01 | 0.00 | 28.00 |
| **CPP** | 370 | 10.34 | 5.55 | 2.00 | 41.00 | 9.88 | 5.51 | 0.00 | 32.00 | 2.14 | 2.46 | 0.00 | 20.00 | 7.72 | 4.98 | 0.00 | 35.00 |
| **QSP** | 200 | 4.58 | 3.70 | 0.00 | 15.00 | 6.68 | 3.65 | 0.00 | 20.00 | 2.07 | 1.37 | 0.00 | 6.00 | 1.89 | 2.12 | 0.00 | 9.00 |
| **SBP** | 80 | 5.60 | 4.65 | 0.00 | 20.00 | 9.33 | 5.17 | 3.00 | 24.00 | 3.99 | 2.72 | 0.00 | 12.00 | 2.71 | 2.28 | 0.00 | 12.00 |

**Table S3** - Feature importance for the predictive model of Anti-Angiogenic peptides.

| **Feature** | **Tool** | **Importance** |
| --- | --- | --- |
| KF1 | Peptides package | 0.058 |
| Z1 | Peptides package | 0.040 |
| hydrophobicity | Peptides package | 0.039 |
| netCharge | Peptides package | 0.034 |
| BLOSUM1 | Peptides package | 0.031 |
| CTDD_charge.3.residue75 | iFeature | 0.028 |
| ROSM880102_mean | Peptides package | 0.028 |
| Z3 | Peptides package | 0.027 |
| KF4 | Peptides package | 0.026 |
| CTDD_polarizability.3.residue75 | iFeature | 0.026 |
| BLOSUM9 | Peptides package | 0.024 |
| WOLS870101_mean | Peptides package | 0.024 |
| CTDD_hydrophobicity_ARGP820101.2.residue25 | iFeature | 0.023 |
| ZIMJ680104_var | AAINDEX | 0.023 |
| CTDD_polarizability.1.residue25 | iFeature | 0.023 |
| BLOSUM10 | Peptides package | 0.023 |
| CTDD_solventaccess.3.residue100 | iFeature | 0.023 |
| WOLR810101_mean | Peptides package | 0.022 |
| CTDD_charge.1.residue50 | iFeature | 0.022 |
| CTDD_polarizability.1.residue0 | iFeature | 0.021 |
| CTDD_hydrophobicity_ZIMJ680101.2.residue100 | iFeature | 0.021 |
| NISK860101_mean | Peptides package | 0.021 |
| CTDD_polarizability.2.residue75 | iFeature | 0.020 |
| ZIMJ680104_mean | Peptides package | 0.020 |
| CTDD_hydrophobicity_PONP930101.1.residue100 | iFeature | 0.019 |
| CTDD_polarizability.3.residue0 | iFeature | 0.019 |
| NADH010104_mean | Peptides package | 0.019 |
| CTDD_hydrophobicity_FASG890101.3.residue75 | iFeature | 0.018 |
| BLOSUM7 | Peptides package | 0.018 |
| WILM950102_mean | Peptides package | 0.018 |
| ROBB760105_mean | Peptides package | 0.018 |
| CTDD_hydrophobicity_PONP930101.1.residue50 | iFeature | 0.017 |
| CTDD_hydrophobicity_ENGD860101.2.residue100 | iFeature | 0.017 |
| NAKH900107_mean | Peptides package | 0.017 |
| CTDD_secondarystruct.2.residue0 | iFeature | 0.017 |
| PONP800102_var | AAINDEX | 0.017 |
| CTDD_charge.1.residue25 | iFeature | 0.017 |
| RACS820109_mean | Peptides package | 0.016 |
| NADH010102_var | AAINDEX | 0.016 |
| VHEG790101_var | AAINDEX | 0.016 |
| OOBM850104_var | AAINDEX | 0.015 |
| CTDD_solventaccess.1.residue50 | iFeature | 0.015 |
| ROSM880101_var | AAINDEX | 0.015 |
| CTDD_solventaccess.2.residue25 | iFeature | 0.015 |
| WILM950104_mean | Peptides package | 0.012 |

**Table S4** - Feature importance for the predictive model of Anti-Bacterial peptides.

| **Feature** | **Tool** | **Importance** |
| --- | --- | --- |
| DISORDER_LONG | Peptides package | 0.064 |
| SST_COIL | Peptides package | 0.053 |
| netCharge | Peptides package | 0.042 |
| KF8 | Peptides package | 0.033 |
| Z4 | Peptides package | 0.025 |
| KF6 | Peptides package | 0.025 |
| BLOSUM1 | Peptides package | 0.020 |
| BLOSUM10 | Peptides package | 0.020 |
| BLOSUM3 | Peptides package | 0.020 |
| Z2 | Peptides package | 0.018 |
| bomanIndex | Peptides package | 0.018 |
| Z1 | Peptides package | 0.015 |
| KF2 | Peptides package | 0.014 |
| NISK860101_mean | Peptides package | 0.013 |
| KF4 | Peptides package | 0.013 |
| KF1 | Peptides package | 0.013 |
| CTDD_hydrophobicity_FASG890101.2.residue25 | iFeature | 0.013 |
| Z3 | Peptides package | 0.013 |
| CTDD_hydrophobicity_FASG890101.3.residue75 | iFeature | 0.013 |
| NADH010102_mean | Peptides package | 0.012 |
| PARJ860101_mean | Peptides package | 0.012 |
| NADH010103_mean | Peptides package | 0.012 |
| WOLS870101_mean | Peptides package | 0.011 |
| NADH010104_mean | Peptides package | 0.011 |
| RADA880101_mean | Peptides package | 0.011 |
| CTDD_hydrophobicity_ARGP820101.1.residue100 | iFeature | 0.011 |
| CTDD_polarity.1.residue0 | iFeature | 0.011 |
| CTDD_hydrophobicity_PRAM900101.3.residue0 | iFeature | 0.010 |
| CTDD_hydrophobicity_ENGD860101.1.residue0 | iFeature | 0.010 |
| PONP800107_mean | Peptides package | 0.010 |
| WOLR810101_mean | Peptides package | 0.010 |
| ROSM880102_mean | Peptides package | 0.010 |
| VHEG790101_mean | Peptides package | 0.009 |
| CTDD_hydrophobicity_ENGD860101.1.residue25 | iFeature | 0.009 |
| mw | Peptides package | 0.009 |
| BLOSUM2 | Peptides package | 0.009 |
| PONJ960101_mean | Peptides package | 0.009 |
| CTDD_hydrophobicity_ZIMJ680101.1.residue75 | iFeature | 0.009 |
| TSAJ990101_mean | Peptides package | 0.009 |
| KF10 | Peptides package | 0.008 |
| CTDD_hydrophobicity_CASG920101.1.residue75 | iFeature | 0.008 |
| OOBM770105_mean | Peptides package | 0.008 |
| CTDD_solventaccess.1.residue25 | iFeature | 0.008 |
| WOLS870102_mean | Peptides package | 0.008 |
| CTDD_normwaalsvolume.2.residue100 | iFeature | 0.008 |
| CTDD_polarity.3.residue25 | iFeature | 0.008 |
| RADA880103_mean | Peptides package | 0.008 |
| CTDD_charge.2.residue50 | iFeature | 0.008 |
| CTDD_polarity.3.residue100 | iFeature | 0.008 |
| CTDD_polarity.3.residue75 | iFeature | 0.008 |
| CTDD_hydrophobicity_ENGD860101.2.residue25 | iFeature | 0.008 |
| ZIMJ680104_mean | Peptides package | 0.008 |
| CTDD_hydrophobicity_PONP930101.1.residue75 | iFeature | 0.007 |
| ZIMJ680104_var | AAINDEX | 0.007 |
| CTDD_solventaccess.3.residue50 | iFeature | 0.007 |
| CTDD_secondarystruct.1.residue100 | iFeature | 0.007 |
| CTDD_polarizability.3.residue75 | iFeature | 0.007 |
| CTDD_secondarystruct.2.residue100 | iFeature | 0.007 |
| ZHOH040102_var | AAINDEX | 0.007 |
| RICJ880115_var | AAINDEX | 0.007 |
| ZHOH040101_var | AAINDEX | 0.007 |
| ROBB760103_mean | Peptides package | 0.007 |
| ROBB760109_mean | Peptides package | 0.007 |
| CTDD_secondarystruct.2.residue50 | iFeature | 0.007 |
| CTDD_charge.2.residue0 | iFeature | 0.007 |
| RACS820109_mean | Peptides package | 0.007 |
| NAKH900101_mean | Peptides package | 0.006 |
| NAKH920103_mean | Peptides package | 0.006 |
| RADA880105_var | AAINDEX | 0.006 |
| ROBB760102_var | AAINDEX | 0.006 |
| ROBB760104_var | AAINDEX | 0.006 |
| NAKH920106_var | AAINDEX | 0.006 |
| RACS820114_var | AAINDEX | 0.006 |
| NADH010104_var | AAINDEX | 0.006 |
| ROBB760101_mean | Peptides package | 0.006 |
| PONP800101_var | AAINDEX | 0.006 |
| RACS820114_mean | Peptides package | 0.006 |
| ROBB760104_mean | Peptides package | 0.006 |
| CTDD_polarity.2.residue25 | iFeature | 0.006 |
| CTDD_secondarystruct.1.residue75 | iFeature | 0.006 |
| PONP800102_var | AAINDEX | 0.006 |
| ROBB760112_var | AAINDEX | 0.006 |
| WILM950103_var | AAINDEX | 0.006 |
| YUTK870103_var | AAINDEX | 0.006 |
| KSCTriad_g1.g2.g1.gap0 | iFeature | 0.005 |
| SUEM840102_var | AAINDEX | 0.005 |
| NADH010107_var | AAINDEX | 0.005 |
| NAKH920107_var | AAINDEX | 0.005 |
| NAKH920103_var | AAINDEX | 0.005 |
| WOLS870103_var | AAINDEX | 0.005 |
| WILM950103_mean | Peptides package | 0.005 |
| F6 | Peptides package | 0.005 |
| CTDD_charge.2.residue100 | iFeature | 0.004 |

**Table S5** - Feature importance for the predictive model of Anti-Cancer peptides.

| **Feature** | **Tool** | **Importance** |
| --- | --- | --- |
| Z3 | Peptides package | 0.07 |
| CTDD_secondarystruct.3.residue100 | iFeature | 0.07 |
| CTDD_solventaccess.3.residue0 | iFeature | 0.04 |
| KF4 | Peptides package | 0.04 |
| hydrophobicity | Peptides package | 0.04 |
| bomanIndex | Peptides package | 0.03 |
| aIndex | Peptides package | 0.03 |
| KF1 | Peptides package | 0.03 |
| CTDD_hydrophobicity_FASG890101.2.residue25 | iFeature | 0.03 |
| Z2 | Peptides package | 0.03 |
| NAKH900111_mean | Peptides package | 0.03 |
| BLOSUM6 | Peptides package | 0.03 |
| ROSM880102_mean | Peptides package | 0.03 |
| NAKH920108_var | AAINDEX | 0.02 |
| KF3 | Peptides package | 0.02 |
| CTDD_hydrophobicity_ZIMJ680101.1.residue25 | iFeature | 0.02 |
| CTDD_polarity.2.residue50 | iFeature | 0.02 |
| PONP800107_mean | Peptides package | 0.02 |
| CTDD_hydrophobicity_ARGP820101.1.residue0 | iFeature | 0.02 |
| RADA880101_mean | Peptides package | 0.02 |
| NAKH900105_mean | Peptides package | 0.02 |
| CTDD_hydrophobicity_ZIMJ680101.1.residue100 | iFeature | 0.02 |
| ZHOH040103_mean | Peptides package | 0.02 |
| WOEC730101_mean | Peptides package | 0.02 |
| WILM950101_mean | Peptides package | 0.02 |
| WOLR810101_mean | Peptides package | 0.02 |
| BLOSUM2 | Peptides package | 0.02 |
| PRAM900101_mean | Peptides package | 0.02 |
| RADA880105_mean | Peptides package | 0.02 |
| CTDD_hydrophobicity_FASG890101.3.residue50 | iFeature | 0.02 |
| CTDD_hydrophobicity_ZIMJ680101.3.residue50 | iFeature | 0.02 |
| WOLS870101_var | AAINDEX | 0.02 |
| NADH010105_var | AAINDEX | 0.02 |
| WILM950103_mean | Peptides package | 0.02 |
| ROBB760107_var | AAINDEX | 0.02 |
| WILM950103_var | AAINDEX | 0.02 |
| CTDD_solventaccess.1.residue25 | iFeature | 0.02 |
| YUTK870102_var | AAINDEX | 0.01 |

**Table S6** - Feature importance for the predictive model of Anti-Viral peptides.

| **Feature** | **Tool** | **Importance** |
| --- | --- | --- |
| SST_COIL | Peptides package | 0.074 |
| CTDD_hydrophobicity_CASG920101.3.residue100 | iFeature | 0.033 |
| BLOSUM1 | Peptides package | 0.026 |
| Z1 | Peptides package | 0.022 |
| aIndex | Peptides package | 0.022 |
| CTDD_hydrophobicity_PRAM900101.3.residue25 | iFeature | 0.021 |
| SST_SHEET | Peptides package | 0.020 |
| CTDD_secondarystruct.1.residue25 | iFeature | 0.019 |
| CTDD_polarizability.3.residue0 | iFeature | 0.019 |
| BLOSUM8 | Peptides package | 0.019 |
| CTDD_secondarystruct.1.residue0 | iFeature | 0.019 |
| BLOSUM7 | Peptides package | 0.019 |
| CTDD_charge.1.residue50 | iFeature | 0.019 |
| Z4 | Peptides package | 0.018 |
| CTDD_hydrophobicity_FASG890101.3.residue25 | iFeature | 0.018 |
| BLOSUM6 | Peptides package | 0.018 |
| BLOSUM3 | Peptides package | 0.017 |
| Z3 | Peptides package | 0.017 |
| KF10 | Peptides package | 0.017 |
| BLOSUM4 | Peptides package | 0.017 |
| CTDD_polarity.1.residue50 | iFeature | 0.017 |
| CTDD_hydrophobicity_FASG890101.2.residue75 | iFeature | 0.017 |
| CTDD_polarizability.2.residue25 | iFeature | 0.016 |
| CTDD_normwaalsvolume.1.residue50 | iFeature | 0.016 |
| CTDD_hydrophobicity_CASG920101.1.residue25 | iFeature | 0.016 |
| instability | Peptides package | 0.015 |
| Z5 | Peptides package | 0.015 |
| CTDD_charge.3.residue100 | iFeature | 0.015 |
| KF6 | Peptides package | 0.015 |
| CTDD_polarizability.2.residue50 | iFeature | 0.015 |
| CTDD_hydrophobicity_ARGP820101.2.residue0 | iFeature | 0.014 |
| CTDD_hydrophobicity_PONP930101.1.residue0 | iFeature | 0.014 |
| CTDD_hydrophobicity_ZIMJ680101.2.residue25 | iFeature | 0.014 |
| CTDD_hydrophobicity_ARGP820101.2.residue25 | iFeature | 0.014 |
| BLOSUM10 | Peptides package | 0.014 |
| WOEC730101_mean | Peptides package | 0.013 |
| ROBB760105_var | AAINDEX | 0.013 |
| NADH010104_mean | Peptides package | 0.013 |
| PARJ860101_mean | Peptides package | 0.013 |
| ROBB760101_mean | Peptides package | 0.013 |
| WOLS870101_mean | Peptides package | 0.013 |
| CTDD_hydrophobicity_ENGD860101.1.residue75 | iFeature | 0.013 |
| ROBB760113_mean | Peptides package | 0.012 |
| ZHOH040103_mean | Peptides package | 0.012 |
| PONP800107_mean | Peptides package | 0.012 |
| YUTK870102_mean | Peptides package | 0.012 |
| NAKH900107_mean | Peptides package | 0.012 |
| NADH010106_var | AAINDEX | 0.012 |
| WOEC730101_var | AAINDEX | 0.012 |
| NAKH920108_mean | Peptides package | 0.012 |
| ROBB790101_var | AAINDEX | 0.012 |
| OOBM850102_var | AAINDEX | 0.011 |
| WOLR790101_var | AAINDEX | 0.011 |
| NAKH900109_mean | Peptides package | 0.011 |
| WOLR810101_mean | Peptides package | 0.011 |
| OOBM770105_mean | Peptides package | 0.011 |
| RADA880105_var | AAINDEX | 0.011 |
| YUTK870103_mean | Peptides package | 0.011 |
| ZIMJ680103_var | AAINDEX | 0.011 |
| NADH010104_var | AAINDEX | 0.010 |
| NAKH920106_var | AAINDEX | 0.010 |
| WOLS870103_var | AAINDEX | 0.010 |
| RADA880106_mean | Peptides package | 0.010 |

**Table S7** - Feature importance for the predictive model of Anti-Inflammatory peptides.

| **Feature** | **Tool** | **Importance** |
| --- | --- | --- |
| CTDD_charge.2.residue0 | iFeature | 0.271 |
| CTDD_hydrophobicity_CASG920101.1.residue0 | iFeature | 0.082 |
| MONM990201_var | AAINDEX | 0.058 |
| CTDD_charge.3.residue75 | iFeature | 0.057 |
| NAKH920102_mean | Peptides package | 0.050 |
| mw | Peptides package | 0.040 |
| CTDD_solventaccess.2.residue0 | iFeature | 0.037 |
| CTDD_secondarystruct.2.residue0 | iFeature | 0.037 |
| BLOSUM1 | Peptides package | 0.036 |
| NAKH920106_var | AAINDEX | 0.036 |
| RACS820113_mean | Peptides package | 0.036 |
| CTDD_hydrophobicity_ZIMJ680101.2.residue100 | iFeature | 0.034 |
| CTDD_polarizability.1.residue75 | iFeature | 0.032 |
| ROBB760104_var | AAINDEX | 0.030 |
| F4 | Peptides package | 0.029 |
| CTDD_hydrophobicity_ARGP820101.1.residue50 | iFeature | 0.029 |
| CTDD_hydrophobicity_ZIMJ680101.3.residue25 | iFeature | 0.028 |
| ROBB760107_var | AAINDEX | 0.028 |
| CTDD_hydrophobicity_FASG890101.3.residue0 | iFeature | 0.027 |
| WILM950103_var | AAINDEX | 0.022 |

**Table S8** - Feature importance for the predictive model of Cell-Penetrating peptides.

| **Feature** | **Tool** | **Importance** |
| --- | --- | --- |
| CTDD_charge.3.residue0 | iFeature | 0.082 |
| WOEC730101_var | AAINDEX | 0.078 |
| KSCTriad_g5.g5.g5.gap0 | iFeature | 0.078 |
| RADA880101_mean | Peptides package | 0.073 |
| NAKH920108_mean | Peptides package | 0.044 |
| PONJ960101_mean | Peptides package | 0.035 |
| NAKH900109_mean | Peptides package | 0.035 |
| NAKH920105_mean | Peptides package | 0.033 |
| ZHOH040103_mean | Peptides package | 0.025 |
| BLOSUM5 | Peptides package | 0.024 |
| bomanIndex | Peptides package | 0.021 |
| PARJ860101_var | AAINDEX | 0.021 |
| CTDD_solventaccess.2.residue100 | iFeature | 0.020 |
| RADA880106_var | AAINDEX | 0.019 |
| ROBB760102_var | AAINDEX | 0.018 |
| NISK860101_mean | Peptides package | 0.014 |
| ONEK900102_var | AAINDEX | 0.014 |
| aIndex | Peptides package | 0.014 |
| CTDD_hydrophobicity_FASG890101.1.residue75 | iFeature | 0.014 |
| TSAJ990101_var | AAINDEX | 0.013 |
| KF2 | Peptides package | 0.013 |
| DISORDER_LONG | Peptides package | 0.013 |
| ROBB760101_mean | Peptides package | 0.013 |
| ROBB760105_mean | Peptides package | 0.012 |
| ROSM880101_var | AAINDEX | 0.012 |
| NADH010106_mean | Peptides package | 0.011 |
| ROBB760112_var | AAINDEX | 0.011 |
| NAKH900109_var | AAINDEX | 0.010 |
| CTDD_normwaalsvolume.1.residue50 | iFeature | 0.010 |
| NAKH900111_var | AAINDEX | 0.009 |
| WOLS870102_mean | Peptides package | 0.009 |
| mw | Peptides package | 0.009 |
| CTDD_charge.3.residue75 | iFeature | 0.009 |
| BLOSUM6 | Peptides package | 0.008 |
| OOBM770105_var | AAINDEX | 0.008 |
| NAKH920101_var | AAINDEX | 0.008 |
| KF9 | Peptides package | 0.007 |
| PARJ860101_mean | Peptides package | 0.007 |
| NADH010105_var | AAINDEX | 0.006 |
| ROSM880101_mean | Peptides package | 0.006 |
| ROBB760111_var | AAINDEX | 0.006 |
| YUTK870104_var | AAINDEX | 0.006 |
| PONP800101_var | AAINDEX | 0.006 |
| CTDD_hydrophobicity_FASG890101.2.residue75 | iFeature | 0.005 |
| NADH010107_mean | Peptides package | 0.005 |
| YUTK870104_mean | Peptides package | 0.005 |
| ROBB760109_var | AAINDEX | 0.005 |
| CTDD_polarity.3.residue100 | iFeature | 0.005 |
| ROBB760111_mean | Peptides package | 0.004 |
| ROSG850101_var | AAINDEX | 0.004 |
| CTDD_hydrophobicity_ENGD860101.3.residue25 | iFeature | 0.004 |
| CTDD_normwaalsvolume.2.residue75 | iFeature | 0.003 |
| PONP800102_var | AAINDEX | 0.003 |
| CTDD_charge.2.residue0 | iFeature | 0.003 |
| CTDD_polarizability.2.residue100 | iFeature | 0.003 |
| TSAJ990102_var | AAINDEX | 0.003 |
| PTIO830102_var | AAINDEX | 0.003 |
| RADA880104_var | AAINDEX | 0.003 |
| CTDD_polarizability.3.residue25 | iFeature | 0.003 |
| hydrophobicity | Peptides package | 0.003 |
| CTDD_polarizability.2.residue25 | iFeature | 0.003 |
| ROBB760108_var | AAINDEX | 0.003 |
| OOBM850105_mean | Peptides package | 0.003 |
| WILM950104_var | AAINDEX | 0.002 |
| CTDD_normwaalsvolume.1.residue75 | iFeature | 0.002 |
| WOLR810101_mean | Peptides package | 0.002 |
| ZIMJ680103_var | AAINDEX | 0.002 |
| ROBB760105_var | AAINDEX | 0.002 |
| NAKH920108_var | AAINDEX | 0.002 |
| OOBM850101_var | AAINDEX | 0.002 |
| CTDD_hydrophobicity_ARGP820101.1.residue0 | iFeature | 0.002 |
| DISORDER_SHORT | Peptides package | 0.002 |
| NAKH920106_var | AAINDEX | 0.002 |
| TANS770107_var | AAINDEX | 0.002 |
| CTDD_hydrophobicity_ENGD860101.1.residue50 | iFeature | 0.002 |
| NAKH920105_var | AAINDEX | 0.002 |
| CTDD_normwaalsvolume.2.residue0 | iFeature | 0.001 |
| CTDD_hydrophobicity_ZIMJ680101.3.residue25 | iFeature | 0.001 |
| NAKH920106_mean | Peptides package | 0.001 |
| CTDD_hydrophobicity_ENGD860101.1.residue100 | iFeature | 0.001 |
| RACS820114_var | AAINDEX | 0.001 |
| TAKK010101_var | AAINDEX | 0.001 |
| CTDD_charge.3.residue25 | iFeature | 0.001 |
| ONEK900101_var | AAINDEX | 0.001 |
| ZHOH040102_var | AAINDEX | 0.001 |
| NAKH920102_var | AAINDEX | 0.001 |
| YUTK870102_mean | Peptides package | 0.001 |
| ROBB760101_var | AAINDEX | 0.001 |
| WARP780101_mean | Peptides package | 0.001 |
| SUEM840102_mean | Peptides package | 0.001 |
| NAKH920104_mean | Peptides package | 0.001 |
| RACS820113_var | AAINDEX | 0.001 |

**Table S9** - Feature importance for the predictive model of Quorum Sensing peptides.

| **Feature** | **Tool** | **Importance** |
| --- | --- | --- |
| CTDD_charge.2.residue0 | iFeature | 0.234 |
| CTDD_charge.1.residue25 | iFeature | 0.110 |
| PONJ960101_mean | Peptides package | 0.079 |
| CTDD_hydrophobicity_ZIMJ680101.3.residue0 | iFeature | 0.077 |
| netCharge | Peptides package | 0.066 |
| ZIMJ680104_mean | Peptides package | 0.054 |
| NADH010106_var | AAINDEX | 0.051 |
| CTDD_hydrophobicity_FASG890101.1.residue75 | iFeature | 0.046 |
| CTDD_charge.2.residue100 | iFeature | 0.045 |
| KF2 | Peptides package | 0.041 |
| CTDD_charge.1.residue100 | iFeature | 0.039 |
| KSCTriad_g2.g2.g2.gap0 | iFeature | 0.024 |
| WILM950101_mean | Peptides package | 0.015 |
| ZIMJ680105_var | AAINDEX | 0.015 |
| NAKH900111_var | AAINDEX | 0.015 |
| CTDD_hydrophobicity_ENGD860101.1.residue100 | iFeature | 0.013 |
| CTDD_hydrophobicity_CASG920101.2.residue75 | iFeature | 0.013 |
| KF4 | Peptides package | 0.010 |
| CTDD_polarity.1.residue100 | iFeature | 0.009 |
| CTDD_charge.1.residue0 | iFeature | 0.008 |
| CTDD_hydrophobicity_FASG890101.1.residue50 | iFeature | 0.008 |
| NADH010102_var | AAINDEX | 0.007 |
| ROBB760112_var | AAINDEX | 0.006 |
| OOBM770104_mean | Peptides package | 0.005 |
| ROBB760104_mean | Peptides package | 0.004 |
| CTDD_hydrophobicity_ARGP820101.1.residue100 | iFeature | 0.004 |
| TAKK010101_var | AAINDEX | 0.002 |
| CTDD_secondarystruct.2.residue100 | iFeature | 0.001 |

**Table S10** - Feature importance for the predictive model of Surface Binding peptides.

| **Feature** | **Tool** | **Importance** |
| --- | --- | --- |
| BLOSUM2 | Peptides package | 0.074 |
| BLOSUM7 | Peptides package | 0.046 |
| Z1 | Peptides package | 0.044 |
| CTDD_hydrophobicity_PONP930101.1.residue50 | iFeature | 0.044 |
| KF1 | Peptides package | 0.043 |
| BLOSUM1 | Peptides package | 0.042 |
| KF4 | Peptides package | 0.042 |
| CTDD_hydrophobicity_CASG920101.1.residue50 | iFeature | 0.041 |
| Z5 | Peptides package | 0.041 |
| CTDD_polarizability.3.residue100 | iFeature | 0.038 |
| ROSM880101_mean | Peptides package | 0.037 |
| NISK860101_mean | Peptides package | 0.037 |
| CTDD_normwaalsvolume.2.residue75 | iFeature | 0.036 |
| NADH010103_mean | Peptides package | 0.034 |
| BLOSUM6 | Peptides package | 0.034 |
| CTDD_hydrophobicity_FASG890101.3.residue25 | iFeature | 0.033 |
| bomanIndex | Peptides package | 0.032 |
| CTDD_polarity.3.residue75 | iFeature | 0.032 |
| ROBB760103_var | AAINDEX | 0.032 |
| OOBM770105_var | AAINDEX | 0.031 |
| CTDD_hydrophobicity_PRAM900101.2.residue75 | iFeature | 0.031 |
| ROBB760101_mean | Peptides package | 0.031 |
| CTDD_hydrophobicity_ZIMJ680101.2.residue0 | iFeature | 0.031 |
| NADH010107_mean | Peptides package | 0.031 |
| NAKH900101_mean | Peptides package | 0.030 |
| RACS820114_mean | Peptides package | 0.027 |
| RADA880103_mean | Peptides package | 0.025 |

**Table S11** - Performance on 10-fold cross validation for training predictive models for eight different therapeutic peptide classes.

| **Peptide Class** | **# features** | **Algorithm** | **AUC** | **TPR** | **TNR** | **F1** | **MCC** |
| --- | --- | --- | --- | --- | --- | --- | --- |
| AAP | 45 | ET | 0.87 | 0.74 | 0.94 | 0.84 | 0.70 |
| ABP | 93 | ET | 0.99 | 0.96 | 0.99 | 0.97 | 0.95 |
| ACP | 39 | ET | 0.97 | 0.93 | 0.96 | 0.94 | 0.89 |
| AIP | 20 | GB | 0.83 | 0.68 | 0.88 | 0.80 | 0.58 |
| AVP | 63 | ET | 0.94 | 0.90 | 0.87 | 0.89 | 0.77 |
| CPP | 92 | XGBOOST | 0.97 | 0.90 | 0.98 | 0.94 | 0.88 |
| QSP | 28 | XGBOOST | 0.98 | 0.97 | 0.97 | 0.97 | 0.94 |
| SBP | 27 | ET | 0.84 | 0.71 | 0.88 | 0.79 | 0.60 |

**Table S12** - Proportion of identical/similar peptides in training and test sets for all peptide classes. Similarity is measured using the SequenceMatcher module, available in the *difflib* Python package, under different cutoffs of similarity.

| **Class** | **75%** | **85%** | **95%** |
| --- | --- | --- | --- |
| AAP | 0.00 | 0.00 | 0.00 |
| ABP | 0.00 | 0.00 | 0.00 |
| ACP | 0.00 | 0.00 | 0.00 |
| AIP | 0.00 | 0.00 | 0.00 |
| AVP | 0.18 | 0.15 | 0.09 |
| CPP | 0.00 | 0.00 | 0.00 |
| QSP | 0.05 | 0.00 | 0.00 |
| SBP | 0.00 | 0.00 | 0.00 |

**Table S13** - Performance of predictive models for ABP, ACP and AVP classes on blind-test sets of peptides retrieved from DRAMP database.

| **Peptide Class** | **Method** | **TPR** | **TP** | **FN** | **F1** |
| --- | --- | --- | --- | --- | --- |
| ABP | CSM-peptides | 0.83 | 2,585 | 524 | 0.90 |
| ACP | CSM-peptides | 0.61 | 61 | 38 | 0.76 |
| AVP | CSM-peptides | 0.63 | 111 | 65 | 0.77 |

**Table S14** - Distribution of training and test sets for the eight classes of peptides in the dataset. Data is divided into a Training set, used to build the predictive models, and two non-redundant test sets.

|  | **Training set** | | **Non-redundant Test set 1** | | **Non-redundant Test set 2** | |
| --- | --- | --- | --- | --- | --- | --- |
| **Peptide Class** | **Positive** | **Negative** | **Positive** | **Negative** | **Positive** | **Negative** |
| AAP | 107 | 107 | 28 | 28 | 28 | 2000 |
| ABP | 800 | 800 | 199 | 199 | 200 | 2000 |
| ACP | 250 | 250 | 82 | 82 | 82 | 2000 |
| AIP | 1258 | 1887 | 420 | 629 | 420 | 2000 |
| AVP | 407 | 544 | 60 | 45 | 60 | 2000 |
| CPP | 370 | 370 | 92 | 92 | 92 | 2000 |
| QSP | 200 | 200 | 20 | 20 | 20 | 2000 |
| SBP | 80 | 80 | 24 | 24 | 24 | 2000 |

**Table S15** - Classes of features calculated for each peptide sequence in the dataset.

| **Feature Class** | **Description** | **Package** | **# Features** |
| --- | --- | --- | --- |
| AAC | Amino acid composition | iFeature | 20 |
| DPC | Dipeptide composition | iFeature | 400 |
| GAAC | Grouped amino acid composition | iFeature | 5 |
| GDPC | Grouped dipeptide composition | iFeature | 25 |
| GTPC | Grouped tripeptide composition | iFeature | 125 |
| CTDC | C/T/D composition | iFeature | 39 |
| CTDT | C/T/D transition | iFeature | 39 |
| CTDD | C/T/D distribution | iFeature | 195 |
| CTriad | Conjoint triad | iFeature | 343 |
| KSCTriad | Conjoint k-spaced triad | iFeature | 343 |
| aIndex | Aliphatic index | Peptides | 1 |
| bomanIndex | Potential interaction index | Peptides | 1 |
| netCharge | Sum of the changes of each amino acid | Peptides | 1 |
| fasgaiVectors | Factor Analysis Scales of Generalized Amino Acid Information | Peptides | 6 |
| hydrophobicity | Hydrophobicity index | Peptides | 1 |
| instability | Instability index | Peptides | 1 |
| blosum | BLOSUM62 derived indices | Peptides | 10 |
| cruciani | Cruciani properties | Peptides | 3 |
| kidera | Kidera factors | Peptides | 10 |
| mw | Molecular weight | Peptides | 1 |
| zScales | Z-scales | Peptides | 5 |
